# Efficiency and novelty of using environmental swabs for dry-surface biofilm recovery

**DOI:** 10.1099/acmi.0.000664.v4

**Published:** 2024-02-29

**Authors:** Fergus Watson, Sandra Wilks, John Chewins, Bill Keevil

**Affiliations:** ^1^​ School of Biological Sciences, University of Southampton, Southampton, UK; ^2^​ Bioquell UK, Andover, UK; ^3^​ School of Health Sciences, University of Southampton, Southampton, UK

**Keywords:** *Acinetobacter baumannii*, biofilm, dry-surface biofilm, environmental swab, healthcare-associated infections

## Abstract

Studies on the epidemiology of dry-surface biofilms (DSBs) within healthcare settings have shown an almost universal distribution across frequently touched items. Despite a growing body of evidence for DSBs in hospitals, little attention has been paid to the recovery capacity of techniques used to detect these microbial communities. Biofilms are inherently difficult to remove from surfaces due to adhesive substances within their matrix and may act as sources of infection, but to what extent is largely unknown. In this study, we evaluate the recovery efficiencies of commonly used environmental swabs against DSBs containing 7.24 log_10_ *Acinetobacter baumannii* cm^−2^, using a drip flow reactor and desiccation cycle. Biofilm presence was visually confirmed using episcopic differential interference contrast microscopy combined with epifluorescence and quantified using sonicated viable plate counts. The swab materials used comprised foam, viscose and cotton, all of which were pre-moistened using a buffer solution. The surfaces were vigorously swabbed by each material type and the resultant microbe populations for both swabs and remaining DSBs were quantified. Our results found foam-tipped swabs to be superior, detecting on average 30 % of the original DSB contamination; followed by viscose (6 %) and cotton (3 %). However, no distinct difference was revealed in the concentration of microbes remaining on the surface after swabbing for each swab type, suggesting there is variation in the capacity for each swab to release biofilm-associated micro-organisms. We conclude whilst environmental swabs do possess the ability to detect biofilms on dry surfaces, the reduced efficiencies are likely to cause an underestimation of the microbes present and should be considered during clinical application.

## Data Summary

Four supplementary figures are available with the online version of this article.

## Introduction

Every year, healthcare-associated infections (HAIs) affect millions of hospitalized patients. Those admitted to intensive care units (ICUs) attribute to 25 % of these infections [1, 2]. The vast majority of HAIs originate from the ESKAPE pathogens, a list of six top global antimicrobial-resistant pathogens (*Enterococcus faecium*, *Staphylococcus aureus*, *Klebsiella pneumoniae*, *Acinetobacter baumannii*, *Pseudomonas aeruginosa* and *Enterobacter* species) [3, 4]. Healthcare environments, such as the ICU, are recognized as high-risk areas for the proliferation of multidrug-resistant organisms (MDROs) due to the extensive use of antibiotics, which applies an inherently selective pressure on the microbiome [5, 6].

Transmission of MDROs, and thus HAIs, will readily occur in hospital environments through contact between the patients, healthcare workers and clinical equipment [7–9]. Studies have shown environmental surfaces pose a significant risk to patients developing HAIs upon admission if the prior occupant was known to be colonized by MDROs, in spite of efforts in infection prevention and routine cleaning of surfaces [10, 11]. Nosocomial pathogens have been shown to survive on desiccating hospital surfaces for several months and are suspected of acting as a reservoir for HAIs. Recently, dry biofilms have been found on these types of surfaces and are being recognized as a potential cause for the persistence of MDROs on healthcare surfaces [12–16]. Biofilms are communities of micro-organisms attached to a substrate and surrounded by a protective structure of extracellular polymeric substance (EPS). Microbes residing within a biofilm are phenotypically more tolerant to antimicrobials such as cleaning agents, and can be up to 1000 times less susceptible than their planktonic counterpart [17]. Referred to as dry-surface biofilms (DSBs), these communities have been detected in abundance across surfaces within an ICU and remained viable despite extensive cleaning using bleach-based disinfectants [15]. During dehydration, biofilms increase the production of EPS, which can lead to increased tolerance to disinfectants, as a result many authors postulate DSB could be used to explain why basic hospital cleaning with previously approved efficacy to planktonic organisms is failing to achieve desired results [16, 17].

Environmental monitoring of clinical surfaces is a fundamental requirement to assess the effectiveness of infection-control measures [18]. As no standardized test currently exists, studies monitoring for DSB often result in physical or destructive removal of surfaces from the hospital room, with subsequent microscopy and culture analysis required to confirm biofilm presence [19, 20]. This approach is not common practice nor readily feasible in most settings. Instead, clinicians will use less-accurate alternatives such as culture swabs, bioluminescence or contact agar [8, 21, 22].

Johani *et al.* used next-generation sequencing of *in situ* DSB samples to demonstrate that environmental swabs alone failed to sample the entire microbiome in comparison to destructive sampling [2]. Swabbing only detects planktonic or loosely bound cultures on the surface, and detection efficiencies for environmental swabs of differing tip and substrate materials are well documented. For instance, the foam swabs used by Johani *et al.* are known to exhibit superior recovery of micro-organisms across a vast range of materials, including brushed stainless steel and polypropylene, compared with cotton or nylon alternatives [23]. In contrast, few studies report on these efficiencies against biofilms and, as a consequence, swab results can be deemed unreliable for DSB detection. In this study, we demonstrate the performance of three swab material types (foam, viscose and cotton) against *in vitro* DSB with similar characteristics to those found on clinical surfaces.

## Methods

### Bacterial strains

The bacterial strain used in this study possessed genes capable of expressing drug-resistance mechanisms to the extended-spectrum β-lactamase antibiotic group. The strain used was *A. baumannii* (NCTC 13301). This species was chosen for its ability to form biofilms and known persistence on healthcare surfaces.

### Inoculum preparation

The strain was sub-cultured into 10 ml tryptic soya broth (TSB) (Sigma Aldrich) overnight at 37 °C. The number of c.f.u. ml^−1^ of bacterial suspension was quantified using serial dilutions and incubation on tryptic soya agar (TSA) (Sigma Aldrich) for 24 h at 37 °C.

### Biofilm model

The biofilms were generated in a drip flow reactor (BioSurface Technologies) assembled as per ASTM standard test E2647-13 using 316 stainless steel coupons as the substrate [13]. The coupons were inoculated with 1 ml culture inoculum at a population of 7 to 8 log_10_ c.f.u. and incubated for 6 h at room temperature (≈20 °C), referred to here as the batch phase. The reactor was then tilted to a 10 ° angle to allow sufficient drainage of waste and shear force across the coupon surface. A sterile supply of 5 % TSB solution was initiated by means of a six channel peristaltic pump (Cole Palmer) at a flow rate of ≈0.9 ml min^−1^ per channel for 36–48 h at room temperature, referred to here as the media phase. At the end of the media phase, the coupons were washed three times with sterile water to remove loosely bound or planktonic cells.

### Desiccation model

Swab performance was measured against biofilm in a dry state. A dry-state biofilm uses a previously described method for biofilm dehydration by means of an aquatic air pump (Hailea) passing room air, via a 0.2 µm in-line filter (Fisher Scientific), across the media surface at 3 l min^−1^ in a sealed 0.01 m^3^ container for 48 to 66 h; referred to here as the drying phase [23]. All biofilm coupons were exposed to a single dehydration cycle. The control coupons were removed at this stage and their population and biofilm formation quantified alongside the test coupons once processed.

### Swabs and surfaces tested

**Fig. 1.**
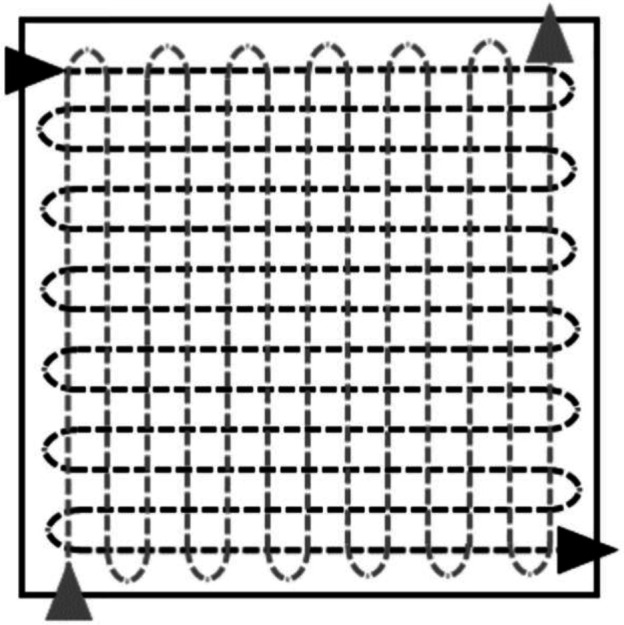
Diagram of the sampling procedure as shown by Jansson *et al.* [30]. The black arrow heads depict the first sweeping motion, whilst the grey arrow heads depict the second time.

Three swab types were used: sterile foam swabs (Technical Service Consultants), sterile viscose swabs (Technical Service Consultants) and sterile cotton swabs (Fisher Scientific). Prior to each experiment, all swabs were sufficiently moistened with phosphate buffer solution (Oxoid); excess liquid was removed by pressing the tip against the tube. Sampling was performed as previously described by sweeping the swab from side to side across the surface whilst rotating the swab, before repeating the process perpendicular to the first sweeping direction (Fig. 1).

### Quantification of swab and coupon population

The log_10_ c.f.u. cm^−2^ for each swab and coupon was quantified in accordance with Johani *et al.*, and ASTM standard test E2647-13 [2, 24]. In brief, for swabs, the tip was aseptically removed into 2 ml PBS and sterile glass beads, and allowed to soak for up to 15 min at room temperature, after which each sample was vortexed twice for 5 s intervals. For the coupons, the surface was scraped and rinsed into 20 ml PBS and sterile glass beads; and the solution homogenized using a vortex twice for 15 s. The number of c.f.u. cm^−2^ for each of the vortexed samples was quantified using serial dilutions and incubation on TSA for 24 h at 37 °C. The number of colonies on each plate was recorded and reported as c.f.u. cm^−2^ using the following calculation:

$$c.f.u./cm^{-2}\;=\;\;\left[\left(\frac{\left(mean\;c.f.u./plate\right)}{volume\;of\;sample\;plated}\right)\;\times \;\left(\frac{volume\;scraped\;into}{surface\;area\;scraped}\right)\;\times \;\left(dilution\right)\;\right]$$

Volume scraped into=2 ml; surface area scraped=18.75 cm^2^.

### Quantification of biofilm colonies

Samples were stained with LIVE/DEAD BacLight bacterial viability kits (Invitrogen); this included both SYTO-9 (green) and propidium iodide (PI) (red). Epifluorescence (EF) microscopy was used to visualize ‘live’ and ‘dead’ bacterial cells, as previously described [23]. ImageJ version 1.52a (National Institutes of Health), with the area measurement tool, was used to determine the percentage area of bioﬁlm in each region of interest (ROI) for swabbed and control coupons [25]. In accordance with Korber *et al.*, a minimum area of 100 000 µm^2^ was analysed at six different areas across the coupon [26]. The datasets generated and analysed during this study are available from the corresponding author upon reasonable request.

### Experimental design

The study consisted of three experimental runs; and within each experimental run, a minimum of two control coupons were used to ensure sufficient DSB present. The same technique and technician was used to conduct all experiments.

### Statistical analysis

The surface loading (c.f.u.) recorded for each coupon was transformed to log_10_ c.f.u. cm^–2^ and all statistical calculations were performed using these values. A Mann–Whitney test was used to compare the quantity of DSB recovered from the processed coupons and swabs with the control; and one-way ANOVA was used to test the statistical significance in the percentage coverage for biofilms quantified during microscopy.

## Results

**Fig. 2.**
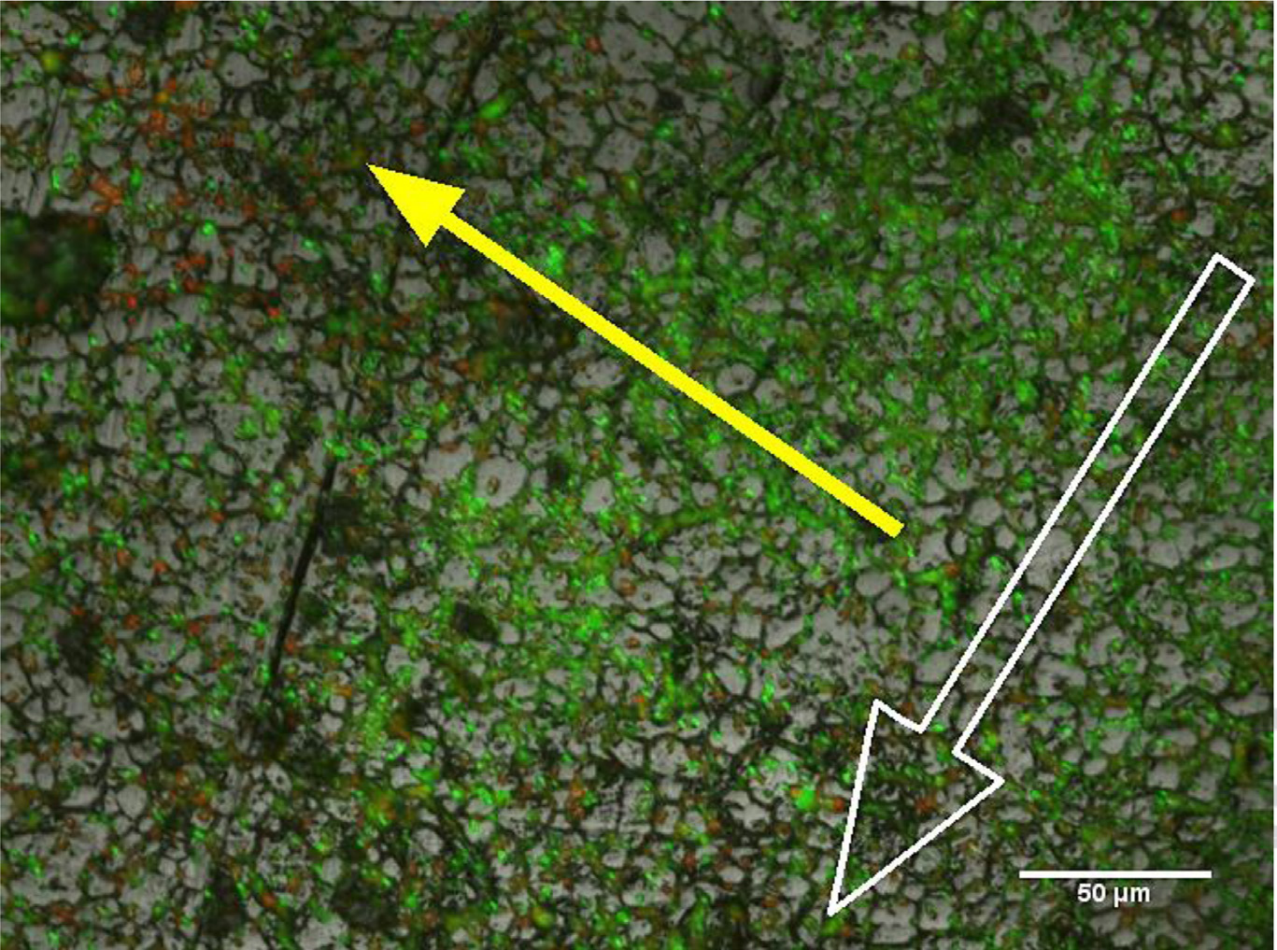
Micrograph of *A. baumannii* DSB taken from the edge of the coupon. The outlined white arrow indicates the direction of flow, whilst the solid yellow arrow indicates a change in microcolony density association with nutrient gradients. There appears to be a great proportion of PI-labelled cells towards the outside of the nutrient gradient versus the centre, which is more abundant in SYTO-9-labelled cells.

Our drip flow reactor model, using a strain of multidrug-resistant *A. baumannii*, successfully generated substantial DSBs with distinct structural features indicative of clinical biofilms as previously described [23]. Episcopic differential interference contrast (EDIC) and EF microscopy highlighted microcolony formation across the topography of the stainless steel (316) substrate surface (Fig. 2). The colonies were seen to aggregate along the cracks and crevices of the steel, representative of those found *in situ* [27]. The density and distribution of biofilm across the surface was shown to vary in relation to the anticipated nutrient gradients, which form along the longitude and latitude axes. This is a unique feature of drip flow reactors due to the directional flow of media over the coupon [24, 28].

Surface bioburden loadings for our control model DSBs averaged 7.24±0.57 log_10_ c.f.u. cm^−2^, which was comparable to worst case scenarios reported on ICU surfaces (7.20 log_10_ c.f.u. cm^−2^) [29]. For this study, the estimated standard deviation for this methodology was 0.23; all DSB control coupons remained within two standard deviations of the mean. There was minimal variation between each experimental run of swab material with a coefficient of variation of 5.80 %, on par with other published data (10.1 %) [23, 29].

Table 1 shows the mean number of *A. baumannii* (c.f.u.) recovered from the surfaces by foam, viscose and cotton swabs, pre-moistened using a buffer solution. There was a statistically significant difference in the number of recovered bacteria for foam swabs in comparison to both viscose and cotton swabs (*P*=0.0094 and *P*=0.0045, respectively). Foam swabs were able to recover up to 18 times more bacteria than the other swab types per surface area sampled. As a result, the microbial load removed by foam swabs demonstrated a closer likeness, in terms of c.f.u., to those of our control coupons (*P*>0.9999); whereas viscose and cotton swabs were significantly lower (*P*<0.0001). As shown in previous studies, foam swabs exhibited superior recovery rates, yet none of the swab types were able to recover more than a third of the total biofilm present [30].

**Table 1.** Data for *A. baumannii* c.f.u. recovered per cm^2^ from a stainless steel coupon using three swab material variants (*n*=9 per swab type) Each experimental run included a minimum of two control coupons, which were exposed to identical desiccating conditions and quantified alongside the test coupons. Recovery rates are given as a percentage of the mean c.f.u. on the control coupons for each run.

Swab	Recovered (log_10_ c.f.u. cm^−2^)	Standard deviation	Recovery rate (%)	Standard deviation
Foam	6.90	0.50	29.79	18.35
Viscose	5.61	0.34	5.75	6.56
Cotton	5.56	0.49	2.87	3.06

Following enumeration of swabbed coupons, the removal rate of surface-bound bacteria averaged 72.28±33.80 % when compared to the total biofilm originally present (Table 2). No statistical difference was observed between the material types for number of bacteria removed (log_10_ c.f.u. cm^−2^) in spite of those recovered from the swab tips as shown above (*P*>0.9999). A comparison between our swabbed and untouched control coupon results indicated that between 38 and 75 % of the microbes removed from the DSBs were unaccounted for. This would imply they had failed to be released from the swab tip during vortexing.

**Table 2.** Data for mean number of *A. baumannii* (c.f.u.) recovered from the surfaces after swabbing (*n*=9 per swab type) All swabbed coupons demonstrated a significant drop in bioburden in comparison to our control coupons (*P*<0.04). Each experimental run included a minimum of two control coupons. Removal rate of bacteria through swabbing was given as a percentage of the mean c.f.u. on the control coupons for each run.

Swab	Swabbed coupons (log_10_ c.f.u. cm^−2^)	Standard deviation	Recovery rate (%)	Standard deviation
Foam	6.57	0.77	67.39	45.43
Viscose	6.42	0.52	80.49	23.77
Cotton	6.71	0.34	70.60	19.72

**Fig. 3.**
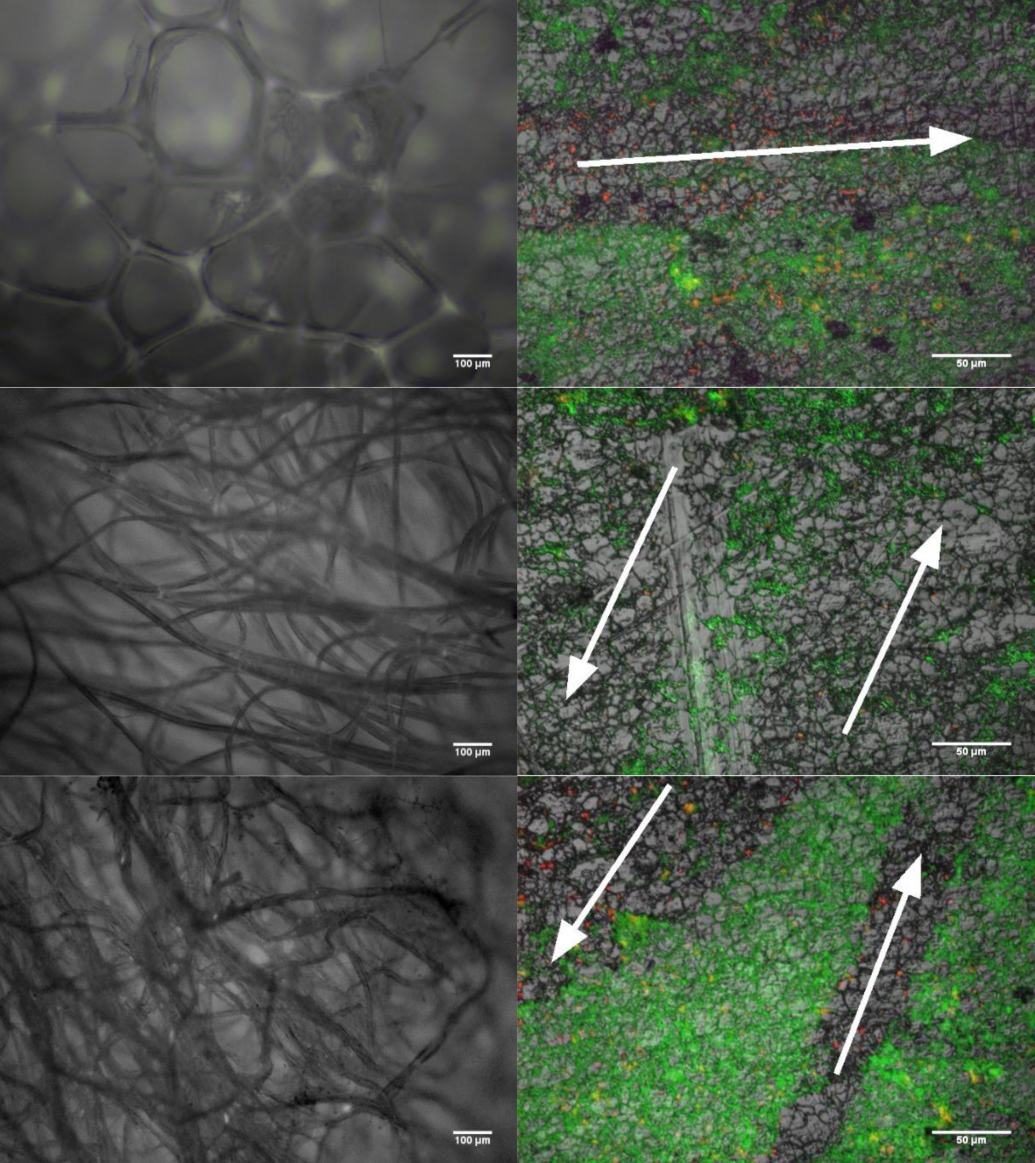
EDIC and EF micrographs of the structure for each swab type (left) and the biofilm surface (right) after being sampled using foam (top), viscose (middle) and cotton (bottom) swabs. The directional arrows (white) indicate the path taken by the swab tip. The direction of flow for the media across the surface was left to right. Traces of dead cells, stained red by PI, can be seen left behind within the path of the swab.

The pronounced differences observed above between swab types were not clearly identified during EDIC and EF microscopy. Using bacterial viability stains SYTO-9 and PI, we could reveal the directional movements of the swab tip across the substrate surface (Fig. 3). Fluorescent staining of microcolonies indicated streak marks visible through the biofilm matrix and the removal of large areas of microcolonies. Within the swabbed areas, we can distinguish non-viable bacteria, stained by PI, left behind within the crevices of the substrate. Areas adjacent to these showed no indication of disruption, suggesting some areas experienced no physical contact with the swab tip.

**Fig. 4.**
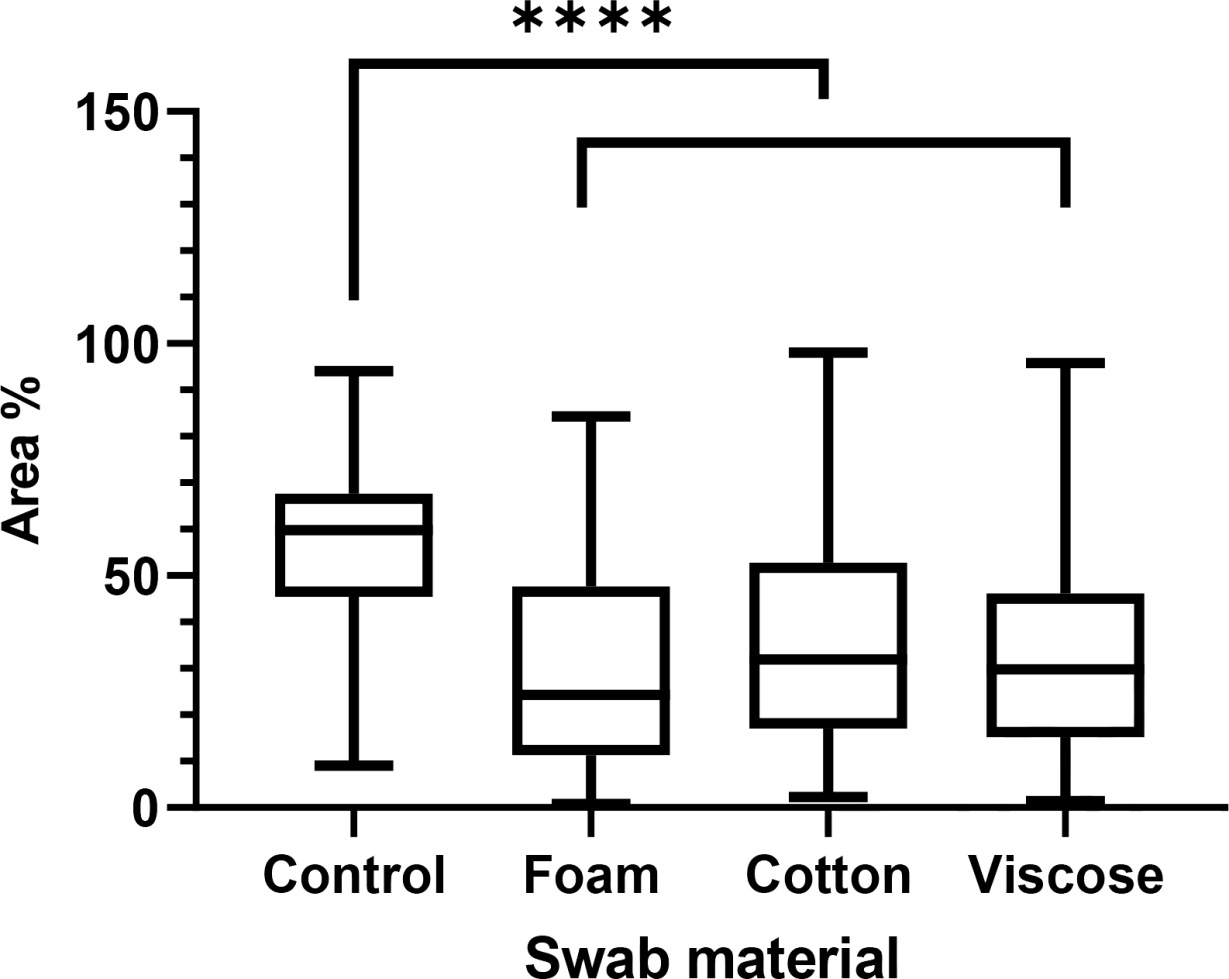
The area of DSB coverage, given as a percentage, remaining on the coupons after swabbing by foam-, viscose- and cotton-tipped swabs. All three swab types demonstrate a reduced level of DSB coverage in comparison with an un-swabbed control (****: *P*<0.0001).

Micrographs of swabbed and un-swabbed coupons, for each swab type, revealed statistically significant reductions in DSB coverage across the coupon, in terms of overall fluorescence per 1000 µm^2^ (*P*<0.0001) (Figs. 1-4). However, no such statistical difference could be distinguished amongst the surfaces swabbed with either foam, viscose or cotton swabs (*P*≥0.0550). This aligns with our c.f.u. values as stated in Table 2 for the same surfaces (Fig. 4).

## Discussion

Patients colonized by epidemiologically important pathogens such as *A. baumannii* will readily shed into their surrounding environment. Studies show, once shed, pathogens can form biofilms and survive on dry surfaces for months and act as reservoirs for future infection [29]. This poses a known risk to newly admitted patients. The same studies highlight the presence of MDROs even when the prior occupant was not a known harbourer, demonstrating the importance of effective surveillance and cleaning [31]. Accurately defining the composition of any environmental microbiome in hospitals is crucial for developing infection prevention and control, such as targeted disinfection [32]. This study has shown surfaces heavily burdened by DSB can be effectively detected using basic environmental swabbing techniques.

The biofilm model used in the study was selected for its unique air–liquid–solid interface deemed indicative of the conditions found on clinical surfaces. The temperature (≈20 °C) at which the model was run, as well as the use of low nutrient media, also reflects the limiting conditions of these surfaces [23]. The heterogenous formation of microcolonies in this model, shown during microscopy, closely resembles *in situ* examples of DSB [14–16]. We observed clusters of viable and non-viable colonies suggesting micro-niche formation in areas of differing nutrient or oxygen concentration. Additionally, the flow of media across the coupon surface introduces a mechanical shear force, which affects the attachment and construction of the biofilm’s matrix such as EPS [33]. We postulate the biofilm’s dense non-uniform formation is more representative of those naturally found in hospitals [20].

Post-swabbing of the surfaces, viable colonies remained embedded within the crevices of the substrate material. We perceive these to be basal cells associated with the base of the biofilm matrix and expressing reduced or no metabolic activity. As a result, this population of cells are often more tolerant of specific antimicrobials [34]. Biofilm adhesion to the substrate is dependent on many factors, including relative humidity and substrate topography. The rate of detachment is generally considered to reduce in the lower layers of biofilm [35–37]. These residual traces of biofilm seen here after swabbing demonstrate a potentially irreversibly bound conditioning film of organic matter allowing rapid recontamination of surfaces – not detected by routine swabs [33, 38].

The impact of swab material on microbe detection is well documented for a vast range of microbes, and materials [30]. Absorbance and subsequent release capacity of a swab is dependent on the properties and architecture of the material, such as flexibility and liquid retention, as well as the osmotic, electrostatic and hydrophobic properties, and cell size of the target micro-organism [39, 40]. For example, foam swabs are designed to have a more flexible and open structure, which enables the swab to sample hard to reach spots and enhanced microbe release during sonication or vortexing. The inverse is true for the tightly woven fibres of cotton swabs [41–43]. We postulate similar performance characteristics can be shown here, whereby foam swabs more accurately identified the total biofilm population present. However, no such difference could be shown for the release capacity across each swab type, where a notable proportion of the DSB removed was not released on any of the swabs tested. Biofilms discovered on similar stainless steel surfaces in food-processing facilities have been found to adhere more readily compared to transient bacteria [44, 45]. Almatroudi *et al.* revealed *in situ* DSB samples taken from an ICU ranged between 420 and 1.60×10^7^ bacteria cm^−2^ [16]. The mean values of DSB recovered in our study by the three swab materials fall within this range (foam=7.94×10^6^ c.f.u. cm^−2^, viscose=4.07×10^5^ c.f.u. cm^−2^, cotton=3.63×10^5^ c.f.u. cm^−2^) and either equal or exceed the group’s published mean value of 5.50×10^5^ bacteria cm^−2^. It is clear from our EF micrographs, in combination with the enumeration values, that the reported bioburden levels for swabbed surfaces were a significant underestimation of the overall contamination present. This reduced efficiency is a known trait when sampling dry surfaces and biofilm formation [25, 46, 47]. The inherent ‘sticky’ nature of polysaccharides, liposaccharides and proteins found in abundance in EPS result in poor release capacities in swabs [33, 48–50].

Published literature shows environmental biofilms are highly abundant on dry surfaces within healthcare facilities – most notably ICUs, where the majority of studies occur. In all the studies, abundance and overall complexity of the biofilms were confirmed by microscopy or culture analysis of surfaces physically removed from the environment. For example, Ledwoch *et al.* (2018) were able to confirm sessile microbes in 95 % of decommissioned equipment samples in spite of negative culture results post-swabbing with sterile cotton swab [20]. Due to the strong surface interactions of DSBs, these studies concluded that microbe surveillance using basic environmental sampling techniques is likely to only collect planktonic organisms [51]. In most circumstances, these are microbes associated with the skin flora readily transmitted by patients and healthcare workers [2, 52]. In the study presented here, we ensured all planktonic or loosely bound microbes were removed prior to processing. Therefore, culture results only reflect the detachment and detection of DSB from the surface and, thus, advocate the use of swabs for detecting environmental biofilms as well as surface-bound micro-organisms in hospitals.

In this study, we were able to demonstrate routine environmental swabs are capable of detecting DSB presence, using a clinically relevant strain and model, at levels similar to those found in healthcare settings. *A. baumannii* was chosen for its ability to form biofilms and importance in HAI. However, it is known DSBs comprise multiple species and future models should incorporate mixed-species biofilms found most frequently, such as *Bacillus* spp. and *S. aureus* [2, 15, 20]. We would anticipate this to have further influence on absorbance and release capacities during swabbing as microbiome composition differs.

The drip flow reactor model described here uses a unique air–liquid–solid interface deemed representative of clinical surfaces, as opposed to other common models where the biofilms are generated on immersed coupons [25, 53, 54]. The low laminar flow of media across the biofilm surface results in comparatively weaker adhesive biofilm on hard surfaces. Based on previous studies, the model used here has a low Reynold’s number (approx. 12–20) [55]. This will have influenced the detachment of DSB fragments from the surface. We acknowledge additional mechanical stresses, such as wiping during cleaning or physical contact from patients, will impact upon the structure of biofilm and its resistance to removal during swabbing. Adaptations of this model should be considered to further support our results.

Environmental sampling, though not mandatory, is used routinely in hospitals to assess surface cleanliness [18, 56]. In part due to the abundance of nutrients used in our study, the average bioburden levels greatly exceed those found *in situ* and the concentration of inoculum per unit of area is an important variable when evaluating sampling methods. Similar swab studies have shown detection efficiencies are directly linked to inoculation levels, with poor recovery rates for lower concentrations, i.e. <10^4^ c.f.u. [40, 55, 57]. Our study highlights good recovery efficiencies for high concentrations (>10^6^ c.f.u) but failed to assess performance through progressively lower levels of bioburden. This could be used to explain why previous DSB studies report poor culture results using swabs.

Biofilm contamination on dry surfaces is rapidly becoming a recognized reservoir for nosocomial pathogens in conjunction with the well-established background in resistance to physical removal and antimicrobials during *in vitro* studies [58]. Developing methods for efficiently detecting surface bioburden such as biofilms is key in combatting outbreaks of HAI pathogens; for example, piezoelectric sensors that utilize a quartz crystal to monitor changes in frequency as mass accumulates on the surface [59]. However, we acknowledge without visualization of surfaces biofilm presence cannot be definitively proven; and basic environmental sampling, as shown here, remains capable of capturing an overall estimation albeit a notable underestimation.

## Supplementary Data

Supplementary material 1
