# Supplementary material for: Efficiency and novelty of using environmental swabs for dry-surface biofilm recovery
Source: Access Microbiol. 2024 Feb 29;6(2):000664.v4. doi: 10.1099/acmi.0.000664.v4 (PMC10928391; doi:10.1099/acmi.0.000664.v4)
Supplement: Supplementary material 1 [file acmi-6-664.v4-s001.pdf]

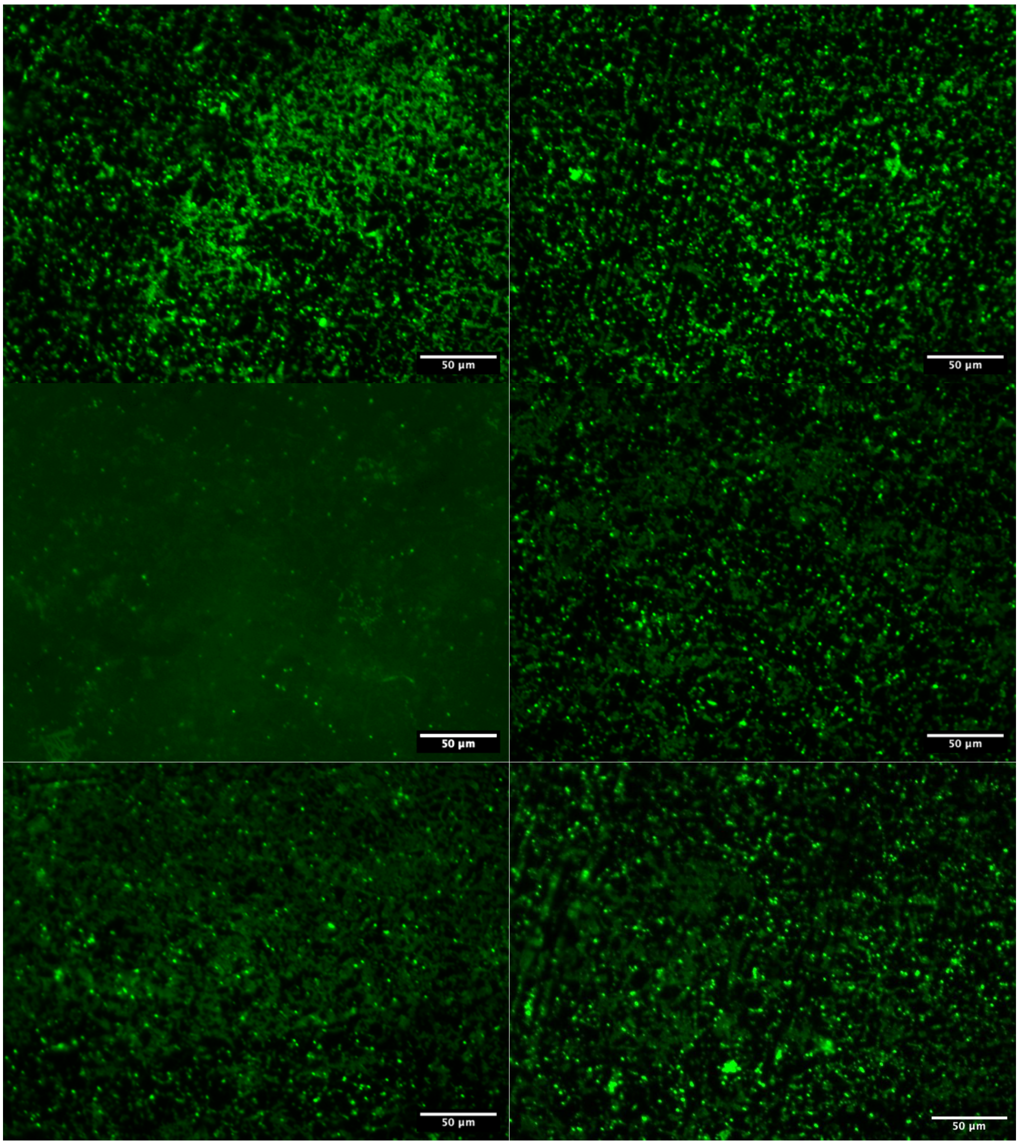

Supplementary Figure 1 –EF micrographs of the un-swabbed biofilm surfaces.

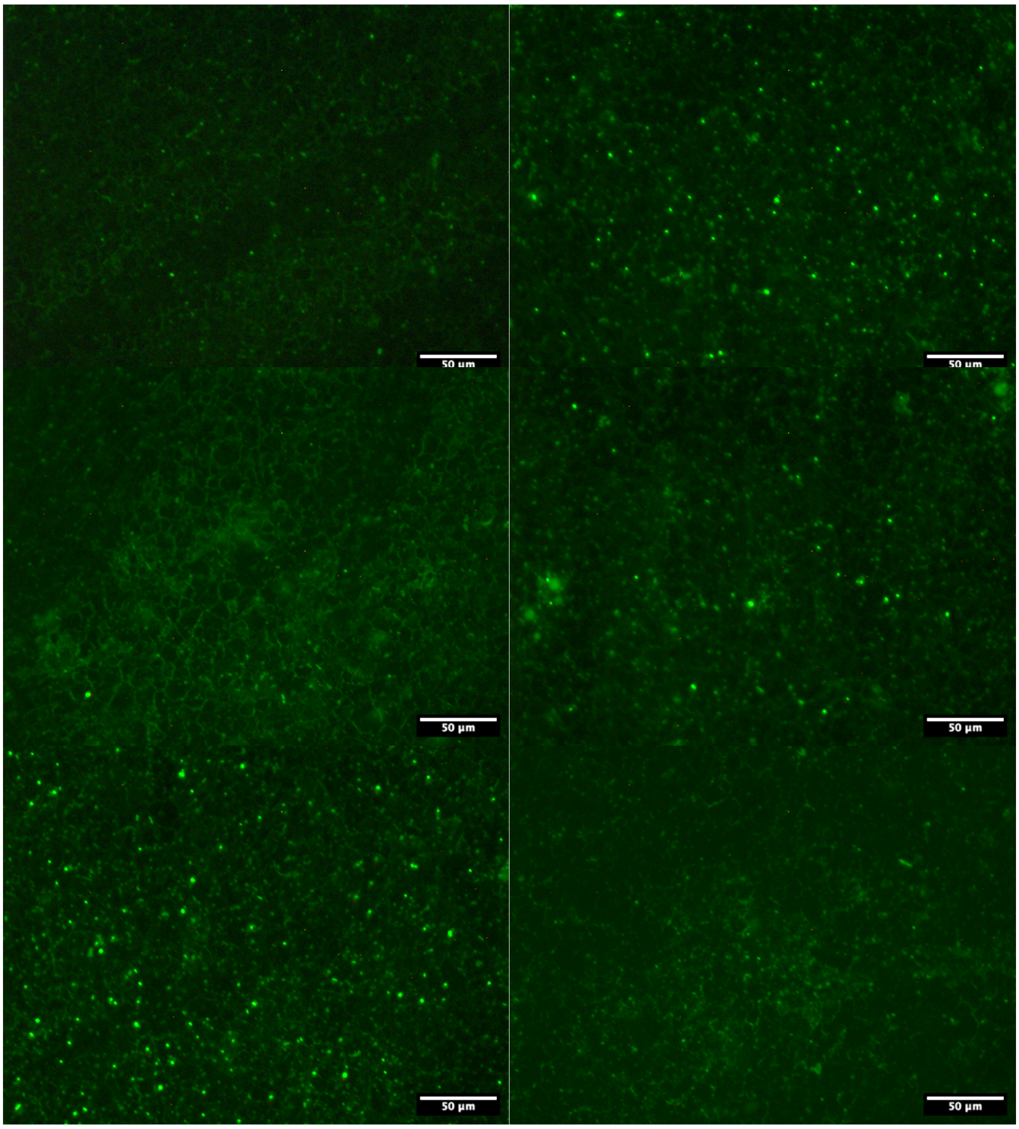

Supplementary Figure 2 –EF micrographs of biofilm after being sampled using foam swabs.

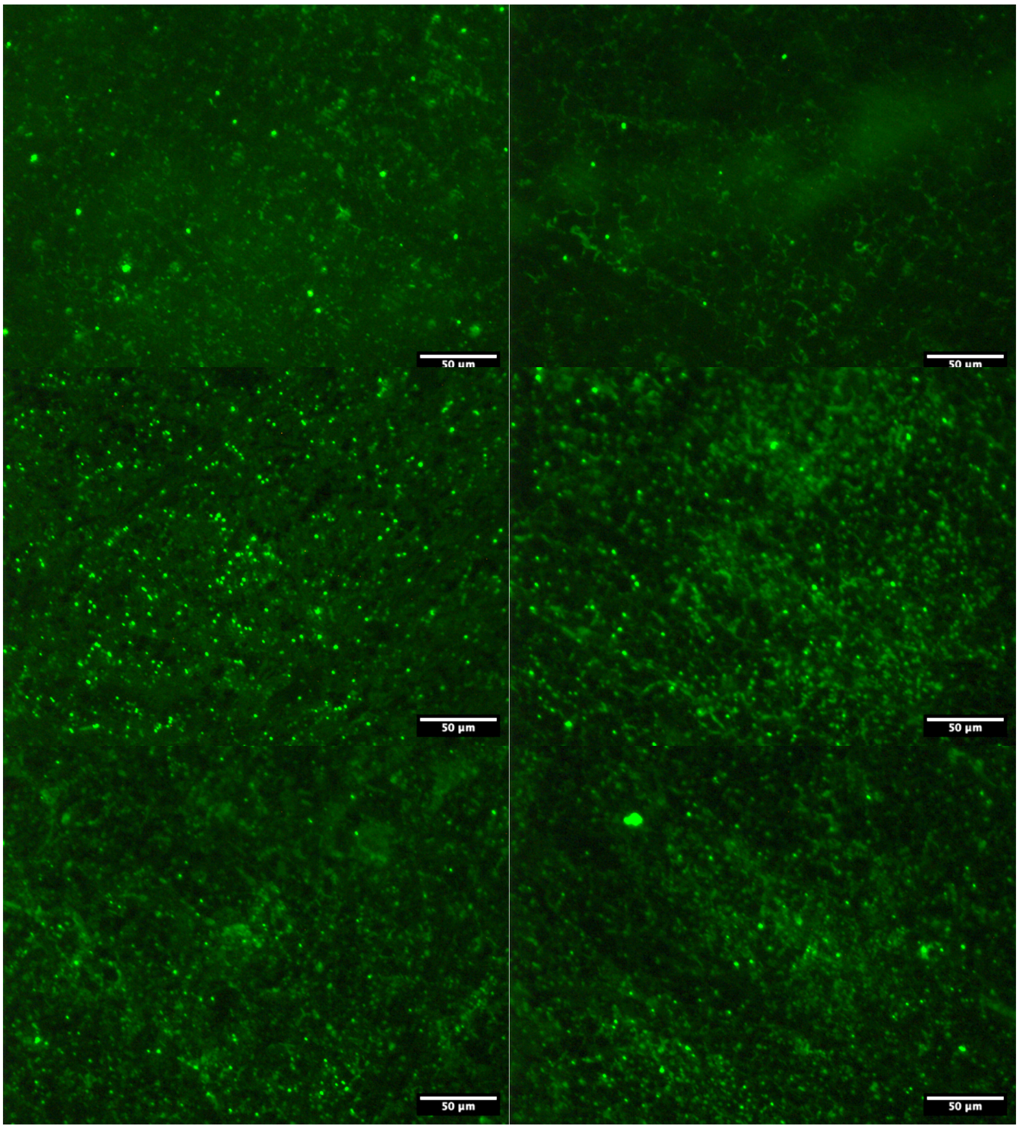

Supplementary Figure 3 –EF micrographs of biofilm after being sampled using cotton swabs.

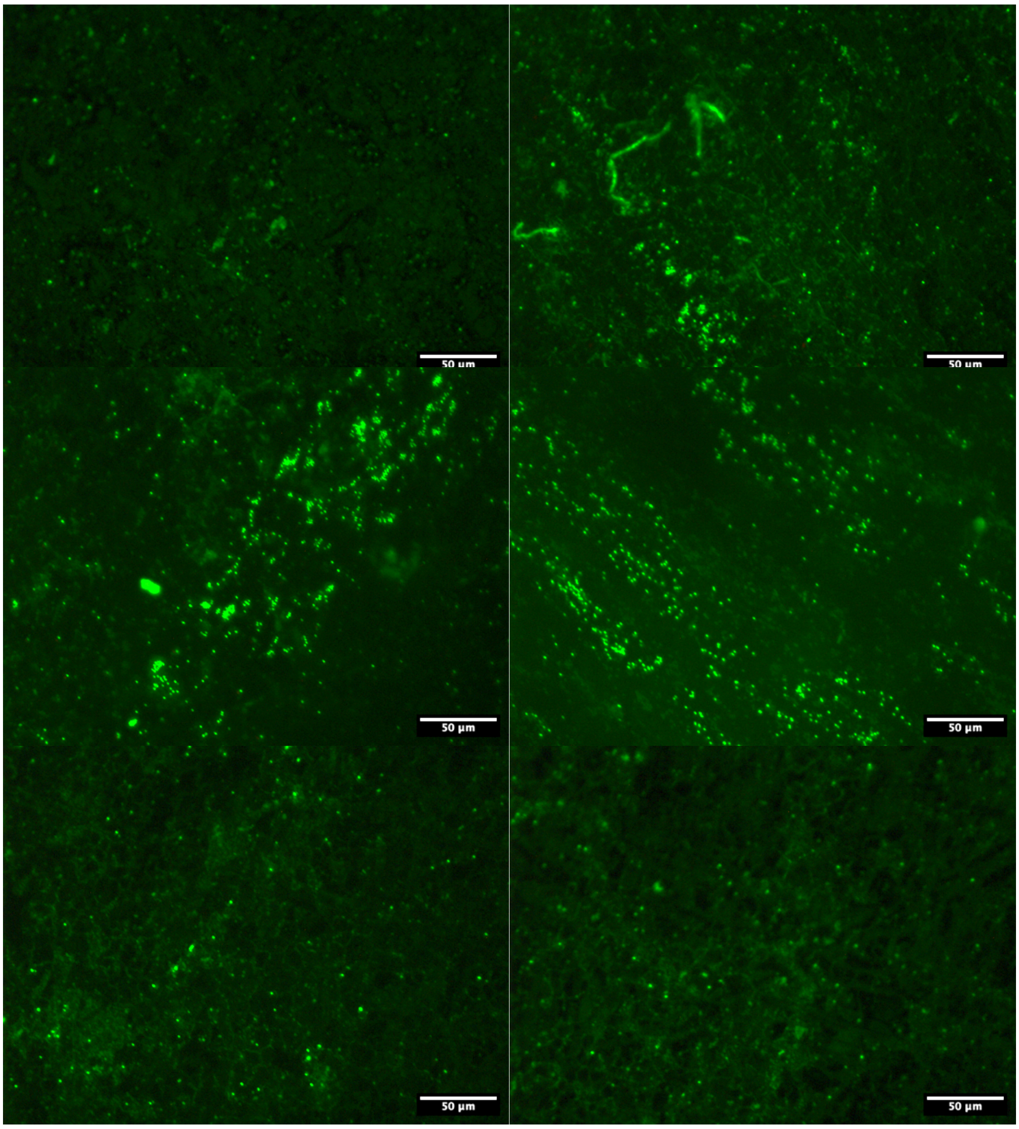

Supplementary Figure 4 –EF micrographs of biofilm after being sampled using viscose swabs.
